# Folic Acid-Targeted Mixed Pluronic Micelles for Delivery of Triptolide

**DOI:** 10.3390/polym16243485

**Published:** 2024-12-13

**Authors:** Meizhen Yin, Xinying Zhang, Tongguang Zhang, Zhiqiang Bao, Zhihui He

**Affiliations:** Medical College, Inner Mongolia Minzu University, Tongliao 028043, China

**Keywords:** triptolide, drug carrier, mixed pluronic micelles, liver cancer, targeted therapy

## Abstract

The present study aimed to explore an ideal delivery system for triptolide (TPL) by utilizing the thin-film hydration method to prepare drug-loaded, folate-modified mixed pluronic micelles (FA–F-127/F-68–TPL). Scanning electron microscopy and atomic force microscopy showed that the drug-loaded micelles had a spherical shape with a small particle size, with an average of 30.7 nm. Cell viability experiments showed that FA–F-127/F-68–TPL significantly reduced HepG2 cell viability, exhibiting strong cytotoxicity. Its cytotoxicity was markedly enhanced compared with bare TPL. Nile red (Nr) was used as a model drug to prepare FA–F-127/F-68–Nr to further validate its tumor-targeting and cellular uptake capability. After coincubation with HepG2 cells, a multifunctional microplate reader showed that intracellular fluorescence intensity significantly increased, indicating that FA–F-127/F-68–Nr could more effectively enter the cells. A nude mouse model of subcutaneous hepatocellular carcinoma was constructed. Following tail vein injection of FA–F-127/F-68–Nr, the fluorescence imaging system showed that FA–F127/F-68–Nr could significantly target tumor tissue, and even if entering the small-sized tumor was challenging, it could be excreted through urine. Nude mice with subcutaneous hepatocellular carcinoma were treated with tail vein injections of FA–F-127/F-68–TPL (45 µg/kg) every other day for 21 days. The results showed that the growth of the transplanted tumors was significantly slowed, with no significant difference compared with bare TPL. In summary, the FA–F-127/F-68–TPL exhibits the advantages of low cost, excellent biological properties, active/passive targeting capabilities, notable cytotoxicity against liver cancer cells, and significant inhibition of transplanted hepatocellular carcinoma growth. Significantly, the FA–F-127/F-68–TPL, despite challenges in targeting tumors with an insignificant EPR effect, can be efficiently excreted via the kidneys, thereby preventing the release of the drug during prolonged circulation and potential damage to normal tissues. Therefore, FA–F-127/F-68–TPL represents a promising antitumor drug delivery system.

## 1. Introduction

Cancer remains a significant threat to human life and health [1]. Despite ongoing advancements in cancer treatment and the emergence of new anti-cancer drugs that have extended patient survival, the overall mortality rate has not seen a substantial decline. Common treatments for malignant tumors include surgical resection, chemotherapy, radiotherapy, and biological therapy, with chemotherapy being pivotal due to its effectiveness in killing cancer cells. However, chemotherapy drugs share several limitations: they have a short half-life, rapidly decreasing blood drug concentrations, low bioavailability, and lack selectivity towards tumor cells. High dosages are necessary, which, while targeting tumors, also severely harm normal tissues. This can lead to serious or long-term toxic side effects that patients must endure. Furthermore, prolonged use of chemotherapy drugs often results in multidrug resistance (MDR) in tumors [2], which can lead to treatment failure. Therefore, addressing tumor drug resistance and reducing the toxic side effects of chemotherapy drugs are critical challenges in current cancer chemotherapy research.

Nanotechnology offers an effective method for targeted drug delivery, opening new avenues for cancer treatment. By employing nanotechnology, small-molecule drugs are encased within nano delivery systems made from natural or synthetic materials, resulting in nano-drug carriers that range in size from 10 to 200 nm. Compared to traditional chemotherapy drugs, these nanocarriers have several advantages [3,4]: they enhance drug solubility and stability, shield drugs from premature degradation, thereby improving pharmacokinetics and extending half-lives, and selectively concentrate drugs in tumor tissues through circulation, improving distribution and significantly increasing drug concentration at the tumor site. This targeted delivery not only reduces dosage but also minimizes the systemic toxic side effects associated with chemotherapy.

Nano-drug carriers leverage their nanoscale size for unique passive targeting in medical applications. Solid tumor tissues, characterized by abundant blood vessels, large endothelial gaps, and limited lymphatic drainage, allow nanoparticles (10–200 nm) to penetrate and accumulate at tumor sites due to the enhanced permeability and retention (EPR) effect [5]. This phenomenon facilitates passive drug accumulation in tumor tissue [6,7]. Additionally, researchers enhance the active targeting capabilities of these carriers by attaching functional groups or active substances that interact specifically with lesions or cell surface receptors. Tumor cells often overexpress receptors like folate, sialoglycan, hyaluronic acid, N-acetylglucosamine, and arginine–glycine–aspartic acid, compared to normal cells [8]. Introducing ligands for these receptors on drug carriers can improve tumor cell endocytosis through receptor-mediated endocytosis [9]. Common targeting ligands include RGD peptides [10], folate [11], transferrin [12], and hyaluronic acid [13], among various antibodies. Modifications with ligands, peptides, and proteins enable nanoparticles to target tumor cells, matrices, and subcellular structures [14,15,16]. Current research aims to develop multifunctional carriers that combine passive and active targeting. These systems adapt to tumor microenvironmental differences, like pH, GSH, and enzyme levels, through pH-sensitive [13,17], redox-sensitive [18], and enzyme-sensitive drug delivery systems [19]. However, multifunctional carriers introduce complexities like increased synthesis steps and costs, complex in vivo behaviors, and potential obstacles due to the added functions. These functions often conflict, especially between enhanced targeting and prolonged circulation, and increase the overall size of the carriers, sometimes exceeding 100 nm, which may reduce permeability within tumor tissues.

Nanocarrier research primarily focuses on liposomes, polymer micelles, peptide-assembled nanoparticles, and inorganic nanoparticles. Liposomes, resembling biological cell membranes with their lipid bilayer structure, encapsulate drugs with low toxicity and immune response and are used in products like erythromycin citrate, vincristine, and triptolide liposomes [20,21,22,23,24]. However, their instability in blood can cause premature drug release, and their larger, insoluble structure makes them prone to clearance by the reticuloendothelial system, reducing efficacy and potentially elevating blood lipids due to their cholesterol content [25,26]. Peptide-assembled nanoparticles are aggregates spontaneously formed by peptide molecules under certain conditions through interactions, possessing specific structures and properties [27]. They present many advantages, including biocompatibility, high drug-loading capacity, chemical diversity, specific targeting, and stimulus-responsive drug delivery [28]. In tumor treatment research, they offer significant advantages [29]. However, their stability and targeting specificity require further optimization, and their toxicity and long-term safety issues need in-depth evaluation. Inorganic nanoparticles, such as gold and silver nanoparticles, carbon nanotubes, graphene oxide, mesoporous silica, and magnetic nanoparticles, offer precise control over size and shape and have unique optical and magnetic properties useful for photothermal therapies [3,30,31,32,33,34]. Despite these advantages, they suffer from poor water solubility and non-biodegradability, remaining in tissues and potentially causing toxicity. Polymer micelles, consisting of a hydrophobic core and a hydrophilic shell, load a broad range of water-insoluble drugs and enhance in vivo transport. Their simple preparation, diverse polymer options, biodegradability, and modifiability make them ideal for cancer treatment research [35]. Typically sized between 10 and 100 nm, they avoid kidney filtration and immune system capture, extending drug circulation times. Mixed micelles, combining polymers like DSPE-PEG with D-α-tocopherol polyethylene glycol succinate (TPGS), offer improved stability, release duration, bioavailability, and drug-loading capacity compared to single polymer systems [36,37,38,39]. Pluronic micelles, low in toxicity and immunogenicity, can reverse tumor multidrug resistance (MDR) and are kidney-excretable, even though they are non-biodegradable [37,40,41]. They have been used to load various chemotherapy drugs such as doxorubicin, paclitaxel, methotrexate, and docetaxel, demonstrating their versatility in drug delivery applications.

Triptolide (TPL) is a diterpenoid extracted from *Tripterygium wilfordii*, known for its anti-inflammatory, immunosuppressive, and antitumor properties. Research has demonstrated TPL’s potent antitumor effects against various cancers in both in vivo and in vitro settings, showing superior efficacy to traditional chemotherapy agents like cisplatin, mitomycin C, and paclitaxel [42]. However, TPL’s lack of tumor selectivity leads to significant toxic side effects and a narrow therapeutic window, limiting its clinical use. To address these issues, recent studies, both domestically and internationally, have explored new drug delivery systems for TPL, including liposomes [43], polymer micelles [44,45], nanoparticles [46], and exosomes [47,48]. These systems have successfully reduced the toxicity associated with TPL, but challenges such as stability, sustained release, tumor tissue penetration, in vivo elimination, bioavailability, and cost of preparation persist, necessitating further improvement and research [12,24,48].

The US Food and Drug Administration has approved Pluronic F-127 and F-68, which are widely utilized in biomedical applications [49]. Folate receptors (FRs) are overexpressed on the surfaces of various tumor cell membranes, including ovarian, endometrial, lung, kidney, breast, and colon cancers, while their expression in normal tissues is minimal [50,51]. Folic acid (FA), a small-molecule vitamin, has a high affinity for these receptors [51,52] and is frequently used in targeted drug delivery systems to enhance the uptake of drug carriers by tumor cells. In this study, we employed the biocompatible Pluronic F-127 and F-68, along with FA, to create FA-modified mixed pluronic micelles loaded with triptolide (TPL) using the thin-film hydration method. Our goal was to develop a drug carrier with optimal physical and biological properties and potent antitumor activity, aiming to establish an effective delivery system for TPL in cancer treatment.

## 2. Materials and Methods

### 2.1. Materials and Reagents

We used pluronic F-127 and F-68 (BASF, Ludwigshafen, Germany), triptolide (purity 98.5%, Aladdin, Shanghai, China), FA (Aladdin), Nile red (Aladdin), fetal bovine serum (FBS; Gibco, Waltham, MA, USA), and Roswell Park Memorial Institute (RPMI) 1640 Medium (Gibco).

### 2.2. Preparation of the Drug Delivery System

Firstly, FA-modified pluronic F127 (FA–F-127) was prepared according to references [53,54]. Subsequently, 200 mg of FA–F-127 and pluronic F-68 (1:1) and 8 mg of TPL drug were dissolved in anhydrous alcohol, and the alcohol solvent was removed to make a drug film. The drug film was hydrated with 15 mL of deionized water at 50 °C and shaken for 30 min. The suspension was transferred to a centrifuge tube and centrifuged at 10,000 rpm for 10 min. The supernatant was taken and filtered through a sterile 0.22 µm cellulose acetate filter to obtain a drug-loaded micelles (FA–F-127/F-68–TPL) solution.

Second, to facilitate the measurement of the active and passive targeting properties of FA–F-127/F-68–TPL, a targeted delivery system for Nile red (Nr), a hydrophobic fluorescent reagent serving as a model drug, was prepared. Using 200 mg of FA–F-127 and pluronic F-68 (1:1) and 8 mg of the Nr model drug, the FA–F-127/F-68–Nr and F-127/F-68–Nr solutions were obtained by the same method as described above.

Third, using 200 mg of FA–F-127 and pluronic F-68 (1:1), without any drug, two blank carrier solutions of FA-F127/F-68 and F-127/F-68 were obtained by the same method as described above.

The TPL content in the FA–F-127/F-68–TPL solutions was determined using reversed-phase high-performance liquid chromatography (RP-HPLC) combined with the standard curve method, and the Nr content in the FA–F-127/F-68–Nr and F-127/F-68–Nr solutions was determined using the UV standard curve method at a maximum absorption wavelength of 553 nm.

### 2.3. Determination of the TPL Loaded with Mixed Pluronic Micelles

The FA–F-127/F-68–TPL and FA–F-127/F-68 solutions were prepared into a freeze-dried powder using the vacuum freezing method. To prepare potassium bromide tablets, FA–F-127/F-68–TPL, FA–F-127/F-68, and TPL were individually mixed with spectral-grade potassium bromide in a mass ratio of 1:100. This mixture was then thoroughly ground in a clean and dried mortar. Subsequently, a Fourier-transform infrared spectrometer (Vertex 80V spectrometer, Bruker, Karlsruhe, Germany) was utilized to scan the spectra of the samples, with excitation wavelengths spanning from 500 to 4000 cm^−1^. The analysis focused on determining the presence of TPL within the mixed pluronic micelles.

### 2.4. Observation of the Morphology and Particle Size Determination of Drug-Loaded Micelles

#### 2.4.1. Scanning Electron Microscope Observation

A small amount of the FA–F-127/F-68–TPL drug-loaded micelle solution was obtained and applied to the conductive tape. After drying and vacuum spraying platinum for 120 s, the morphology of FA–F-127/F-68–TPL was observed, and scanning electron microscope (SEM) images were captured by a scanning electron microscope (JSM-7610F Plus, JEOL, Tokyo, Japan).

#### 2.4.2. Atomic Force Microscopy Observation

After repeatedly diluting the FA–F-127/F-68–TPL drug-loaded micelle solution, a small amount of the sample was obtained and placed on the mica flakes. After drying, it was observed and photographed under an atomic force microscope (AFM, BioScopy Catalyst, Bruker AXS Inc., St. Barbara, CA, USA).

#### 2.4.3. Particle Size Determination

After the prepared drug-loaded micelle solution was diluted, the particle size of FA–F-127/F-68–TPL was measured using a particle size analyzer (Malvern, Worcestershire, UK), with the empty carrier FA-F-127/F-68 as the control.

### 2.5. Cells and Cell Culture

HepG2 cells (human liver cancer cell line) were purchased from the Chinese Type Culture Collection Center of Wuhan University. HepG2 cells were cultured in a cell culture incubator at 37 °C and 5% CO_2_ and routinely cultured in RPMI-1640 medium dcontaining 10% fetal bovine serum for the following cell experiments.

### 2.6. CCK-8 Assay for Cell Viability

HepG2 cells in the logarithmic growth phase were inoculated onto a 96-well culture plate. After wall adherence, the cells were divided into groups, with eight wells per group. Different concentrations of FA–F-127/F-68–TPL groups were replaced with complete culture media containing different concentrations of FA–F-127/F-68–TPL (calculated TPL concentration, 25, 50, 100, and 200 ng/mL). The corresponding concentration of the naked drug TPL groups was replaced with a complete culture medium containing TPL at the corresponding concentration; the blank carrier FA–F-127/F-68 group was replaced with a complete culture medium containing high-concentration FA-F127/F-68, whereas the control group was replaced with a fresh complete culture medium. After cell culture for 24, 48, and 72 h, respectively, the manufacturer’s instructions of the CCK-8 kit were followed, and a multifunctional enzyme-linked immunosorbent assay (SpectraMax Paradigm, Molecular Devices, San Jose, CA, USA) was used to measure the absorbance (OD) of each well of cells at a wavelength of 490 nm. The activity of the cells is obtained by calculating the ratio of the OD value of the experimental group to the OD value of the control group and then multiplying by 100%.

### 2.7. Preparation of Liver Cancer Transplant Tumor Model

Male BALB/c-nu 5–6-week-old nude mice (purchased from the Institute of Experimental Animal Research, Chinese Academy of Medical Sciences, Beijing, China) were housed in SPF-grade laboratories. To prepare a subcutaneous transplantation tumor model of liver cancer, HepG2 cells were amplified and subcutaneously inoculated into the right forelimb of nude mice. Tumor growth was observed daily. The prepared liver cancer transplant tumor model was used for the following tumor-targeting experiments and tumor treatment experiments.

### 2.8. Determination of the Tumor-Targeting of Drug-Loaded Micelles

When the liver cancer-transplanted tumors in nude mice were approximately 80, 120, or 500 mm^3^, the tumor-bearing mice were divided into the FA–F-127/F-68–Nr and control groups (three per group), and the corresponding FA–F-127/F-68–Nr and FA–F-127/F-68 solutions were injected into the tail vein, with 0.2 mL per mouse. When the tumors were approximately 500 mm^3^, the injection concentration increased threefold compared to 120 or 80 mm^3^. Using a fluorescence imaging system (IVIS^®^ Lumina XRMS Series III, PerkinElmer Inc, Waltham, MA, USA) at 0.5 h, 1 h, or 2 h after injection, whole-body fluorescence imaging was performed to observe the distribution of FA–F-127/F-68–Nr in subcutaneous tumor sites, and the tumor-bearing mice were immediately euthanized for fluorescence imaging of isolated organs and tumor tissues to observe the distribution of FA–F-127/F-68–Nr in the isolated heart, liver, spleen, lung, kidney, pancreas, and tumor tissues.

### 2.9. Determination of the Drug Carrier Entering the Tumor Cells

HepG2 cells were inoculated onto a black transparent 96-well plate, and after the cells adhered to the wall, the cells were divided into groups of eight wells each. The cells in the FA–F-127/F-68–Nr group and the F-127/F-68–Nr group were replaced with culture media containing different concentrations of FA–F-127/F-68–Nr and F-127/F-68–Nr, respectively (calculated as Nr; the concentrations were 0.1, 0.2, and 0.4 μg/mL, respectively). The blank carrier group cells were replaced with culture media containing different concentrations of FA–F127/F-68 (the empty micelle solution was diluted to varying magnifications of 10×, 20×, 40×, and 80×) to observe whether the carrier FA–F-127/F-68 produced fluorescence. The control group cells were replaced with fresh complete culture medium to measure the background fluorescence. After cell incubation in the 96-well plate for a certain period, the culture medium was removed, and the HepG2 cells were washed thrice with PBS buffer. Subsequently, 150 μL of DMSO was added to each well, and the plate was shaken at 300 rpm for 15 min. The fluorescence intensity of the cells was measured (excitation wavelength, 552 nm; emission wavelength, 650 nm) using a multifunctional enzyme-linked immunosorbent assay.

### 2.10. Evaluation of the Therapeutic Effect of FA–F-127/F-68–TPL on Liver Cancer Xenografts

When the tumor volume of tumor-bearing mice reached approximately 50 mm^3^, 18 tumor-bearing mice were randomly divided into three groups and treated with tail vein injection for 3 weeks. The mice in the FA–F-127/F-68–TPL and TPL groups were injected with 0.2 mL of FA–F-127/F-68–TPL (dosage based on TPL) and TPL solution, respectively, at a dose of 45 µg/kg every other day. The mice in the model group were injected with an equal volume of physiological saline. ① The long diameter (a) and short diameter (b) of the tumor were measured every 2–3 days; the tumor volume v = ab^2^/2 was calculated, and the tumor growth inhibition rate was calculated as tumor inhibition rate = [model group (dn − do) − administration group (dn − do)]/model group (dn − do) × 100%, where dn is the tumor volume after treatment and do is the tumor volume before treatment. ② The tumor-bearing mice of each group were weighed weekly. ③ After 3 weeks of treatment, the nude mice were euthanized, and the tumor was isolated. The size of the tumor was measured and weighed. ④ The transplanted tumors of each group of tumor-bearing mice were separated to prepare light microscopy specimens based on conventional methods, namely paraffin sectioning and hematoxylin–eosin (HE) staining. An optical microscope was used to observe and photograph the structure of the tumor tissues in each group.

### 2.11. Statistical Analysis

All the experimental data were presented as averages with standard deviation ($\bar{x}\pm s$), and the significance of the mean difference was examined using the Student’s *t*-test.

## 3. Results and Discussion

### 3.1. FA-Modified Pluronic Micelles Loaded with Triptolide

**Figure 1:**
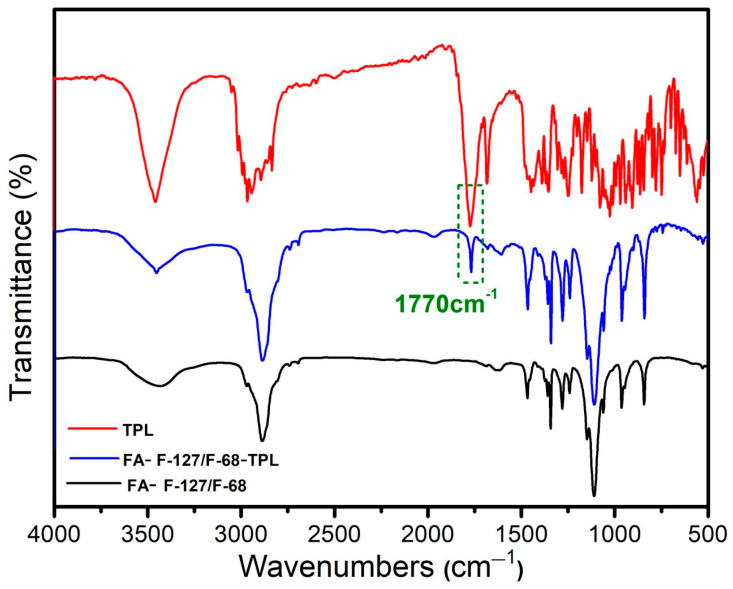
Infrared spectra of FA–F-127/F-68–TPL, FA-F127/F-68, and TPL.

Figure 1 shows the spectra of FA–F-127/F-68–TPL, FA–F-127/F-68, and naked drug TPL determined by infrared spectroscopy. Comparing the spectra of FA–F-127/F-68–TPL and FA-F127/F-68, a new infrared absorption peak was found at 1770 cm^−1^ in the former spectrum, corresponding to the strong absorption peak of TPL at 1770 cm^−1^. This peak is the stretching vibration absorption peak of the carbonyl group (C=O) on the molecule of triptolide. It can be inferred that the prepared drug-loaded micelles are loaded with TPL.

### 3.2. Morphology and Particle Size of the FA–F-127/F-68–TPL

**Figure 2:**
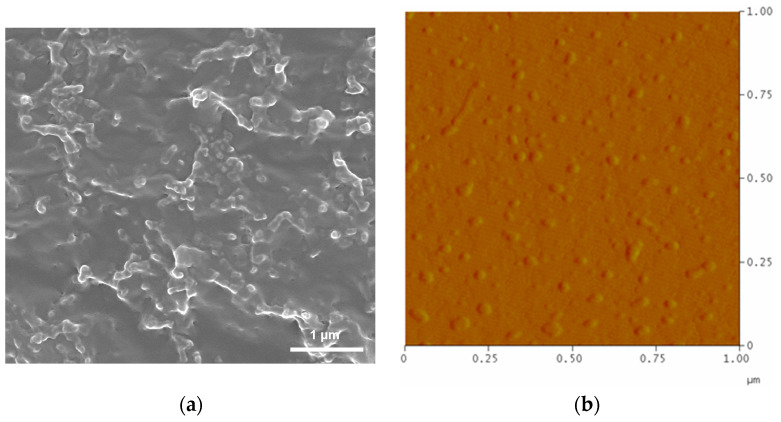
Morphology of the FA–F-127/F-68–TPL drug-loaded micelles: (**a**) SEM images; (**b**) AFM images.

**Figure 3:**
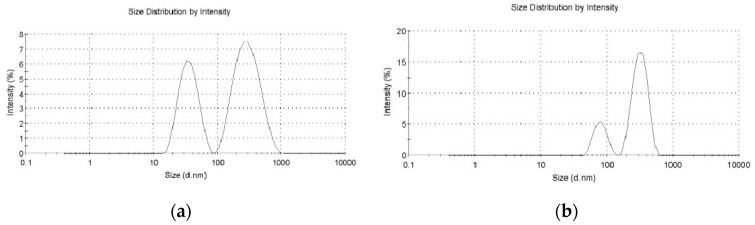
Size distribution of the FA–F-127/F-68–TPL drug-loaded micelles: (**a**) FA–F-127/F-68–TPL; (**b**) FA–F-127/F-68.

SEM and AFM observations showed that the FA–F-127/F-68–TPL drug-loaded micelles were spherical with small particle sizes, generally not exceeding 100 nm (Figure 2a,b). The SEM shows significant agglomeration of colloidal particles (Figure 2a), and the AFM shows good dispersion of colloidal particles (Figure 2b). Figure 3 shows that the particle sizes of FA–F-127/F-68–TPL and FA–F-127/F-68 measured by the particle size analyzer have two peaks, with dispersion indices (PDIs) of 0.574 and 0.404, respectively. In the first peak, the particles of FA–F-127/F-68–TPL accounted for 37.4%, with an average particle size of 36.4 nm and a particle size distribution range of 15.7–78.8 nm; in comparison, particles of FA–F-127/F-68 account for 21.9%, with an average particle size of 80.3 nm and a particle size distribution range of 50.7–122 nm. In the second peak, the average particle size and particle size distribution range for FA–F-127/F-68–TPL were 311 nm and 106–825 nm, respectively, whereas for FA–F-127/F-68, they were 322 nm and 164–531 nm. These results indicate that the average particle size of FA-F-127/F-68-TPL is reduced compared to that of FA–F-127/F-68. SEM and AFM showed that the adhesion between the colloidal particles caused the second peak in the particle size distribution, which may be related to the degree of dilution of the micelles.

Polymer micelles have nano-sized spherical micelles because the hydrophilic segments of the polymer molecules are often larger than the hydrophobic segments, forming conical molecules with “large head groups”. Subsequently, the conical molecules aggregate into the lowest-energy spherical structure through hydrophobic interactions and adsorb the hydrophobic TPL in the hydrophobic core. The micelles formed by amphiphilic block copolymers are relatively stable, but research indicates that the interaction between the hydrophilic and hydrophobic blocks of two different pluronic micelles can increase the stability of the drug delivery system, which is not affected by blood dilution [55]. Thus, the prepared FA–F-127/F-68–TPL drug-loaded micelles are relatively stable, and releasing the drugs prematurely in the blood is difficult. The particle size analyzer measures the particle diameter. Nanoparticles have strong adsorption properties and undergo soft aggregation; the more aggregated the particles, the larger the measured particle size. The aggregation of drug-loaded micelles may make them resistant to dilution in the blood and have better stability in long-term circulation.

Based on literature reports, materials F-127 and F-68 have good biocompatibility [49], mixed micelles are resistant to blood dilution and do not release drugs prematurely [36,37,38,39], and smaller particle-sized drug carriers easily penetrate tumors with high diffusion barriers [56,57]. Nano-drug delivery systems with a particle size of approximately 30 nm can be distributed deep into tumors, and their efficacy is significantly better than similar nanoparticles of approximately 100 nm [58]. The FA–F-127/F-68–TPL prepared in this experiment with an average particle size of 30.7 nm has good biocompatibility, stability, long cycling, and permeability within the tumor.

### 3.3. Cell Toxicity of FA–F-127/F-68–TPL

**Figure 4:**
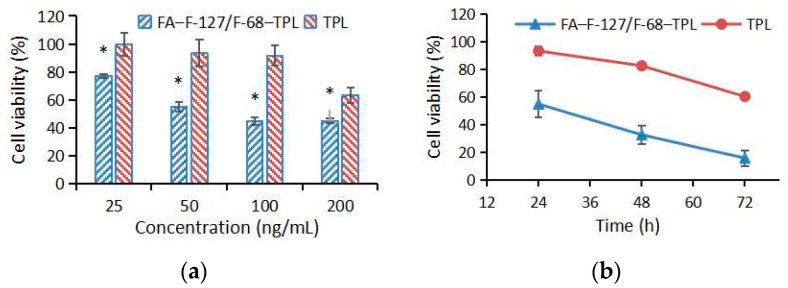
Cell viability of HepG2 cells treated with FA–F-127/F-68–TPL: (**a**) treatment of HepG2 cells with different concentrations of FA–F-127/F-68–TPL for 24 h; (**b**) treatment of HepG2 cells with 100 ng/mL FA–F-127/F-68–TPL for 24, 48, and 72 h. * *p* < 0.01 indicates significant difference.

Lower cell viability reflects stronger drug toxicity. There is no significant difference in the viability of HepG2 cells between the high-concentration blank carrier FA–F-127/F-68 and the control groups (*p* > 0.05), indicating that the FA–F-127/F-68 had no impact on the viability of HepG2 cells. Compared with the control group, FA–F-127/F-68–TPL at 25, 50, 100, and 200 ng/mL, as well as TPL at 100 and 200 ng/mL, significantly reduced cell viability after 24 h of treatment with HepG2 cells (*p* < 0.01), and the effect of FA–F-127/F-68–TPL was significantly enhanced compared with TPL at the same concentration (*p* < 0.01) (Figure 4a). After 24, 48, and 72 h of treatment with 50 ng/mL, the cell viability in both the FA–F-127/F-68–TPL and TPL groups gradually decreased. At different time points, the cell viability in the FA–F-127/F-68–TPL group was significantly lower than that in the TPL group (*p* < 0.01) (Figure 4b). The results indicate that FA–F-127/F-68–TPL exhibits stronger cytotoxicity towards HepG2 cells compared with that of TPL.

### 3.4. Targeting Performance of the FA–F-127/F-68–TPL

**Figure 5:**
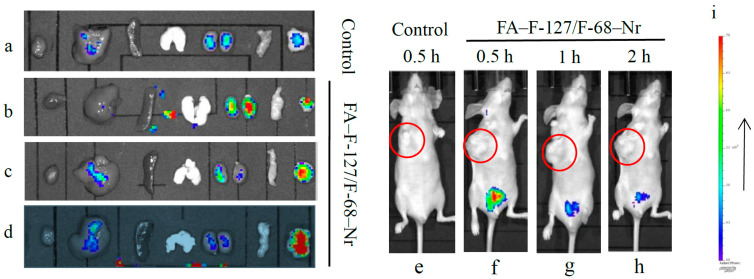
Fluorescent imaging of whole-body and isolated organs of tumor-bearing mice after tail vein injection of FA–F-127/F-68–Nr. Order of organs: heart, liver, spleen, lung, kidney, pancreas, and tumor. (**a**–**d**) Fluorescence imaging of isolated tumor tissues and organs 1 h after injection: (**a**) control group, (**b**–**d**) FA–F-127/F-68–Nr group; tumor volume of approximately (**b**) 80 mm^3^, (**c**) 120 mm^3^, and (**d**) 500 mm^3^. (**e**–**h**) Whole-body fluorescent imaging when the tumor volume is approximately 500 mm^3^, (**e**) control group, 0.5 h after injection. (**f**–**h**) FA–F-127/F-68–Nr group; (**f**) 0.5 h, (**g**) 1 h, and (**h**) 2 h after injection. (**i**) The color from top to bottom indicates the fluorescence intensity level. Red and blue indicate the strongest and weakest fluorescence, respectively.

After 1 h of tail vein injection, no fluorescence was observed at the subcutaneous transplant sites of tumor-bearing mice in both the FA–F-127/F-68–Nr group and the control group. Blue background or no fluorescence was observed in the isolated heart, liver, spleen, lungs, kidneys, pancreas, and tumor tissues in the control group (Figure 5a). In contrast, strong red fluorescence was observed in tumors with volumes of approximately 80, 120, and 500 mm^3^ in the FA–F-127/F-68–Nr group. Except for strong red fluorescence in the kidneys when the tumor volume was approximately 80 mm^3^, background fluorescence was observed in all other organs (Figure 5b–d). When the transplanted tumor volume was approximately 500 mm^3^, at 0.5 h after injection of a higher concentration of FA–F-127/F-68–Nr, strong red fluorescence was observed in the bladder, whereas at 1 and 2 h after injection, weak background fluorescence was noted in the bladder (Figure 5f–h), which may be due to the smaller particle size of FA–F-127/F-68–Nr being excreted through the kidneys during circulation. These results indicate that FA–F-127/F-68–Nr has good tumor targeting when the transplanted tumor volume is approximately 120 and 500 mm^3^. Only when the tumor volume is approximately 80 mm^3^, a large amount of FA–F-127/F-68–Nr is excreted by the kidneys and a small amount targets the tumor tissue.

The passive targeting function of nanomedicine carriers mainly relies on the EPR effect. The EPR effect is only more pronounced in tumors with a volume > 100 mm^3^ [59]. Therefore, FA–F-127/F-68–Nr is less distributed in 80 mm^3^ tumors due to the insignificant EPR effect and more distributed in the 120 and 500 mm^3^ tumors where the EPR effect is more pronounced. For tumors with a volume of 80 mm³, due to the insignificant EPR effect, the passive targeting function of any nanomedicine carrier, including FA–F-127/F-68–Nr, is not easily exerted. Furthermore, nano-drug carriers targeting tumor tissue require long circulation to continuously aggregate at the tumor site. For those nano-drug carriers that are not easily concentrated in tumors with insignificant EPR effects, this undoubtedly extends their circulation time in the bloodstream. Consequently, they are inevitably susceptible to the influence of blood flow shear during this prolonged circulation, which may lead to changes in their particle size or stability. Drug carriers with particle sizes < 10 nm can be excreted by the kidneys through a filtration membrane. FA–F-127/F-68–Nr nanoparticles, characterized by their small particle size, undergo continuous long circulation due to their challenge in extensively accumulating within 80 mm³ tumor tissues. Consequently, these nanoparticles are continually influenced by blood flow, causing their size to diminish further and ultimately facilitating renal excretion. This characteristic represents an advantage compared to some nano-drug carriers, especially those with particle sizes > 100 nm, which experience decreased stability during prolonged circulation, leading to premature drug release and potential damage to normal tissues.

### 3.5. Uptake of FA–F-127/F-68–TPL by Hepatocellular Carcinoma Cells

**Figure 6:**
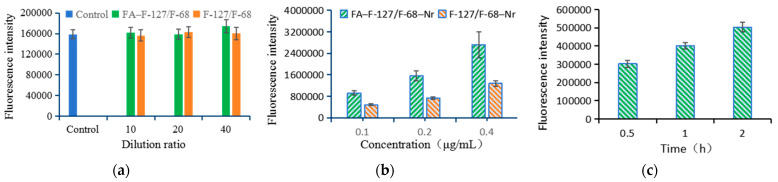
The fluorescence intensity values of HepG2 cells in each group. (**a**,**b**) HepG2 cells coincubated for 2 h with (**a**) FA–F-127/F-68 and F-127/F-68 and (**b**) FA–F-127/F-68–Nr and F-127/F-68–Nr. (**c**) HepG2 cells coincubated with 5 μg/mL FA–F-127/F-68–Nr.

After coincubation with HepG2 cells at 10-, 20-, and 40-fold dilutions of FA–F-127/F-68 for 2 h, intracellular fluorescence intensity was not significantly different between the different concentrations of FA–F-127/F-68 groups (*p* > 0.05). No significant difference was observed in the background fluorescence intensity compared with the control group (*p* > 0.05) (Figure 6a), indicating that the carrier material does not produce background fluorescence. Therefore, subtracting the background fluorescence intensity of cells in the control group from the fluorescence intensity of cells in the FA–F-127/F-68–Nr group can reflect the intracellular content of FA–F-127/F-68–Nr. When FA–F-127/F-68–Nr at concentrations of 0.1, 0.2, and 0.4 μg/mL were coincubated with HepG2 cells for 2 h, the intracellular fluorescence intensity increased with the increased FA–F-127/F-68–Nr concentration, the fluorescence intensity was significantly different between the different concentration groups (*p* < 0.01), and compared with F-127/F-68–Nr at the same concentration, the intracellular fluorescence intensity also significantly increased (*p* < 0.01) (Figure 6b). When 5 μg/mL FA–F-127/F-68–Nr were coincubated with HepG2 cells for 0.5, 1, and 2 h, the intracellular fluorescence intensity increased with prolonged coincubation time (Figure 6c). These results indicate that the higher the concentration of FA–F-127/F-68–Nr or the longer the co-incubation time, the higher the FA–F-127/F-68–Nr content in the cells, indicating a strong endocytosis ability of HepG2 cells toward FA–F-127/F-68–Nr. Moreover, folate ligands enhance the endocytosis ability of HepG2 cells, suggesting that the prepared FA–F-127/F-68–TPL has a good active targeting effect and can quickly deliver drugs to HepG2 cells. Previous literature has confirmed that folate ligands can enhance the endocytosis ability of folate-receptor-positive tumor cells towards drug carriers [60].

### 3.6. FA–F-127/F-68–TPL Inhibits the Growth of Subcutaneous Liver Cancer Xenografts

**Figure 7:**
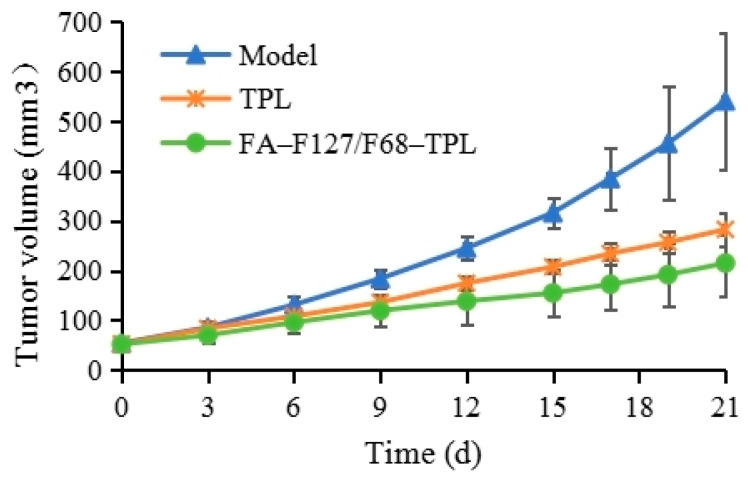
Volume change curves of transplanted tumors in each group treated for 21 days.

After 3 weeks of treatment, the volume changes in the liver cancer-transplanted tumors in each group are shown in the curve (Figure 7). The transplanted tumors in the model group rapidly grew, whereas the transplanted tumors in the FA–F-127/F-68–TPL and TPL groups slowed down significantly. After 7 days of treatment, the transplanted tumor volume in the FA–F-127/F-68–TPL and TPL groups decreased significantly compared with the model group (*p* < 0.01), but the transplanted tumor volume was not significantly different between the two treatment groups (*p* > 0.05). Table 1 shows the inhibition rates of FA–F-127/F-68–TPL on transplanted tumors are higher than those of TPL after 7, 14, and 21 days of treatment, but no significant difference can be observed (*p* > 0.05). After 3 weeks of treatment, the average tumor weight of the FA–F-127/F-68–TPL and TPL groups was significantly lower than that of the model group (*p* < 0.01), but the average tumor weight was not significantly different between the two treatment groups (*p* > 0.05), indicating that FA–F-127/F-68–TPL and TPL have significant inhibitory effects on the growth of liver cancer xenografts, with no significant difference between the two, but the inhibitory effect of the former tends to be greater than that of the latter. Table 2 shows no statistically significant difference in body weight among the tumor-bearing mice within the two treatment groups compared to the model group (*p* > 0.05), suggesting that the doses of FA–F-127/F-68–TPL and TPL used during treatment were safe.

### 3.7. Effect of FA–F-127/F-68–TPL on the Tissue Structure of Liver Cancer

**Figure 8:**
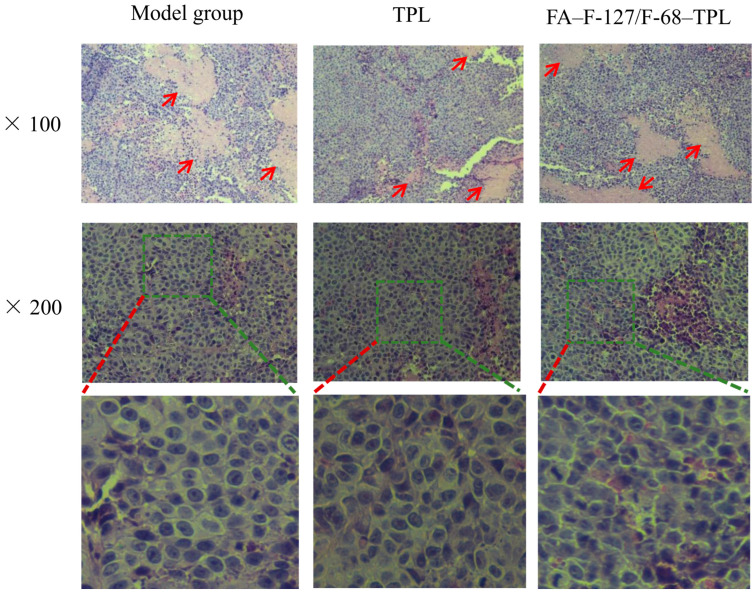
Pathological results of liver cancer-transplanted tumors in the treatment group and model group.

Figure 8 shows the light microscopic structures of the tumor tissues stained with HE from the three groups. The pathological morphology of the transplanted tumors in the model and treatment groups FA–F-127/F-68–TPL and TPL were all epithelioid structures. All three groups had necrosis foci in the tumor tissues, and cancer cells were tightly arranged but disordered in the tumor tissues surrounding the necrotic foci, with a significantly higher proportion of nuclear-cytoplasmic cancer cells. However, the cancer cells in the model group were significantly larger and had clear nucleoli, whereas the nuclei of cancer cells were slightly reduced, and the nucleoli were not obvious in the FA–F-127/F-68–TPL and TPL groups. Particularly, the changes in the nuclei and nucleoli of cancer cells were more obvious, and the nuclear membrane boundaries were unclear in the FA–F-127/F-68–TPL group.

The nucleus and nucleolus are associated with cancer cell proliferation. The changes in the nuclei and nucleoli of cancer cells in the FA–F-127/F-68–TPL and TPL groups reflect a slowed cancer cell proliferation. Due to the rapid proliferation of cancer cells and relatively slow angiogenesis, more necrotic lesions were observed in the tumor tissue of the model group. Studies have shown that TPL can exert antitumor effects through multiple pathways, including inhibiting cancer cell proliferation, inducing cancer cell apoptosis, inducing cancer cell autophagy, blocking the cell cycle, inhibiting cancer cell migration, invasion, and metastasis, reversing multidrug resistance, mediating tumor immunity, and inhibiting angiogenesis [61]. Although cancer cell proliferation was significantly slowed down in the FA–F-127/F-68–TPL and TPL groups, necrotic lesions were also commonly observed in the tumor tissues of both groups, which may be mainly due to the drug inhibition of tumor angiogenesis.

With small tumor size, blood vessels have not yet formed, or the tumor necrosis center has no blood vessels, and the EPR effect of nanomaterials does not exist [62]. The EPR effect is more significant only in tumors with a volume > 100 mm^3^ [59]. Thus, the EPR effect is stronger with abundant blood vessels within the tumor. Therefore, the EPR effect will be weakened if tumor angiogenesis is inhibited. So far, almost all nanomedicine designs have relied on the high permeability of tumor blood vessels to enter the tumor matrix, i.e., targeted delivery relying on the EPR effect [59]. However, most antitumor drugs have inhibitory effects on tumor angiogenesis, which will become a difficult obstacle to overcome for effective targeted drug delivery. TPL can inhibit tumor angiogenesis, thereby affecting its EPR-dependent targeted delivery, which may be the main reason why the antitumor effect of FA–F-127/F-68–TPL in animal experiments is not significantly different from that of the naked drug TPL.

## 4. Conclusions

The prepared FA–F-127/F-68–TPL has several advantages, such as a simple preparation method, low cost, and good biocompatibility. It also has favorable biological properties, especially strong active/passive targeting abilities, which enable it to significantly target liver cancer tissues and enhance the uptake rate by liver cancer cells. FA–F-127/F-68–TPL with smaller particle size not only facilitates better penetration into deep tumor tissues compared to larger drug carriers but also allows renal excretion during prolonged circulation when its targeting toward tumors with unobvious EPR effects has obstacles. This feature presents a unique advantage compared to nano-drug carriers, especially those with particle sizes greater than 100 nm, which may release drugs prematurely during prolonged circulation, potentially damaging normal tissues. Furthermore, FA–F-127/F-68–TPL exhibits significant cytotoxicity against liver cancer cells in vitro, and its toxicity is superior to that of the bare drug TPL. More importantly, FA–F-127/F-68–TPL can also markedly inhibit the growth of liver cancer xenografts. Although the inhibition of intratumoral angiogenesis by the loaded drug itself may affect its passive targeting function, resulting in an enhanced but not statistically significant trend in tumor inhibition compared to the bare drug TPL, FA–F-127/F-68–TPL’s remarkable tumor selectivity significantly outperforms the uncoated TPL, greatly reducing drug-related toxicity and side effects.

## Figures and Tables

**Table 1:** Growth inhibition rate and tumor weight of each group during treatment ($\bar{x}\pm s$, *n* = 6).

Group	Inhibition Rate (%)			Tumor Weight (g)
	7 d	14 d	21 d	21 d
Model group	-	-	-	0.39 ± 0.07
TPL	34.99 ± 24.67	44.93 ± 15.45	52.59 ± 13.75	0.19 ± 0.05 *
FA–F-127/F-68–TPL	48.30 ± 12.36	63.77 ± 6.257	66.63 ± 7.114	0.15 ± 0.02 *

Compared with the model group, * *p* < 0.05.

**Table 2:** Body weight of tumor-bearing mice in each group during treatment ($\bar{x}\pm s$, *n* = 6).

Group	Body Weight (g)		
	7 d	14 d	21 d
Model group	17.94 ± 0.4197	18.41 ± 0.7124	18.86 ± 0.7279
TPL	17.97 ± 1.0667	19.00 ± 0.8659	19.94 ± 1.591
FA–F-127/F-68–TPL	18.27 ± 0.8672	18.63 ± 0.8726	19.67 ± 1.3167

## Data Availability

Data are contained within the article.
